# Draft Genome Sequence of *Cladonia macilenta* KoLRI003786, a Lichen-Forming Fungus Producing Biruloquinone

**DOI:** 10.1128/genomeA.00695-13

**Published:** 2013-09-05

**Authors:** Sook-Young Park, Jaeyoung Choi, Jung A Kim, Min-Hye Jeong, Soonok Kim, Yong-Hwan Lee, Jae-Seoun Hur

**Affiliations:** Korean Lichen Research Institute, Sunchon National University, Suncheon, South Koreaa; Department of Agricultural Biotechnology, Fungal Bioinformatics Laboratory, Center for Fungal Genetic Resources, and Center for Fungal Pathogenesis, Seoul National University, Seoul, South Koreab; Wildlife Genetic Resources Center, National Institute of Biological Resources, Incheon, South Koreac

## Abstract

The lichen-forming fungus *Cladonia macilenta* strain KoLRI003786 is capable of producing an acetylcholinesterase inhibitor, biruloquinone, which effectively prevents neurodegeneration in Alzheimer’s disease. Laying the foundation to unravel the biruloquinone biosynthetic pathway, we present the 37.11-Mb draft genome sequence of strain KoLRI003786.

## GENOME ANNOUNCEMENT

Lichens are remarkable symbionts with a vault of potentially valuable chemical compounds as secondary metabolites. Biruloquinone is one such high-value metabolite produced by lichen-forming fungi and belongs to the *ortho*-phenanthraquinone group. It has been shown to be a potent antioxidant and an effective preventative agent against neurodegeneration in Alzheimer’s disease (AD) (1). Two lichen-forming fungi, *Parmelia birulae* (2) and *Cladonia macilenta* (1), are currently known to produce this chemical. In particular, a mass liquid culture system for the production of biruloquinone by *C. macilenta* has been established (1). However, no information is available on the genes that are responsible for the biosynthesis of biruloquinone in these fungi.

*C. macilenta* strain KoLRI003786 was isolated from rotten bark at Mt. Cangshan (26°31′52.7″N, 100°02′14.0″E), Yunnan Province, China, in 2005. DNA from an axenic culture of the fungus was extracted using the DNeasy mini kit (Qiagen, Valencia, CA). Sequencing was performed using a whole-genome shotgun strategy with Illumina HiSeq 2000 (Macrogen, Inc., Seoul, Korea). The total size of the assembled genome of *C. macilenta* KoLRI003786 is 37,117,081 bp, with a G+C content of 44.68%, representing 540-fold coverage. The short reads were assembled by using Allpaths-LG (3), yielding 240 scaffolds (≥1,000 bp) generated from 1,310 contigs. Gene prediction was performed by using MAKER (4), producing 7,322 protein-coding sequences. According to three gene family pipelines (5–7), 141 transcription factor (TF) genes, 58 cytochrome P450 genes, and 1,362 genes encoding secretory proteins were predicted. In addition, 8 putative polyketide synthase genes, containing ketoacyl synthase, acyltransferase, and acyl carrier domains, were predicted by a domain search (8).

The genome sequence of *C. macilenta* KoLRI003786 is a valuable resource to identify the genes for the biosynthesis of biruloquinone and many other chemicals, and it also serves as a platform to facilitate comparative genomics with other lichen-forming fungi, as well as species in the phylum *Ascomycota*.

### Nucleotide sequence accession numbers.

The draft genome sequence of *C. macilenta* KoLRI003786 has been deposited in DDBJ/EMBL/GenBank under the accession no. AUPP00000000. The version described in this article is the first version, accession no. AUPP01000000.

## References

[1] Luo H Li C Kim JC Liu Y Jung JS Koh YJ Hur JS 2013 Biruloquinone, an acetylcholinesterase inhibitor produced by lichen-forming fungus *Cladonia macilenta*. J. Microbiol. Biotechnol. 23:161–166. 23412057 10.4014/jmb.1207.07016

[2] Krivoshchekova OE Stepanenko LS Mishchenko NP Denisenko VA Maksimov OB 1983 A study of aromatic metabolites of lichens of the family *Parmeliaceae*. II. Pigments. Chem. Nat. Compd. 19:270–274

[3] Gnerre S Maccallum I Przybylski D Ribeiro FJ Burton JN Walker BJ Sharpe T Hall G Shea TP Sykes S Berlin AM Aird D Costello M Daza R Williams L Nicol R Gnirke A Nusbaum C Lander ES Jaffe DB 2011 High-quality draft assemblies of mammalian genomes from massively parallel sequence data. Proc. Natl. Acad. Sci. U. S. A. 108:1513–1518 21187386 10.1073/pnas.1017351108 PMC3029755

[4] Cantarel BL Korf I Robb SM Parra G Ross E Moore B Holt C Sánchez Alvarado A Yandell M 2008 MAKER: an easy-to-use annotation pipeline designed for emerging model organism genomes. Genome Res. 18:188–196 18025269 10.1101/gr.6743907 PMC2134774

[5] Park J Park J Jang S Kim S Kong S Choi J Ahn K Kim J Lee S Kim S Park B Jung K Kim S Kang S Lee YH 2008 FTFD: an informatics pipeline supporting phylogenomic analysis of fungal transcription factors. Bioinformatics 24:1024–1025 18304934 10.1093/bioinformatics/btn058

[6] Choi J Park J Kim D Jung K Kang S Lee YH 2010 Fungal secretome database: integrated platform for annotation of fungal secretomes. BMC Genomics 11:105. 10.1186/1471-2164-11-105 20146824 PMC2836287

[7] Moktali V Park J Fedorova-Abrams ND Park B Choi J Lee YH Kang S 2012 Systematic and searchable classification of cytochrome P450 proteins encoded by fungal and oomycete genomes. BMC Genomics 13:525. 10.1186/1471-2164-13-525 23033934 PMC3505482

[8] Hunter S Jones P Mitchell A Apweiler R Attwood TK Bateman A Bernard T Binns D Bork P Burge S de Castro E Coggill P Corbett M Das U Daugherty L Duquenne L Finn RD Fraser M Gough J Haft D Hulo N Kahn D Kelly E Letunic I Lonsdale D Lopez R Madera M Maslen J McAnulla C McDowall J McMenamin C Mi H Mutowo-Muellenet P Mulder N Natale D Orengo C Pesseat S Punta M Quinn AF Rivoire C Sangrador-Vegas A Selengut JD Sigrist CJ Scheremetjew M Tate J Thimmajanarthanan M Thomas PD Wu CH Yeats C Yong SY 2012 InterPro in 2011: new developments in the family and domain prediction database. Nucleic Acids Res. 40:D306–D312. 10.1093/nar/gkr948 22096229 PMC3245097

